# Does percutaneous dilatational tracheostomy increase the incidence of sternal wound infection - a single center retrospective of 4100 cases

**DOI:** 10.1186/s13019-015-0365-z

**Published:** 2015-11-06

**Authors:** Lachmandath Tewarie, Rachad Zayat, Helga Haefner, Jan Spillner, Andreas Goetzenich, Rüdiger Autschbach, Ajay Moza

**Affiliations:** 1 Department of Thoracic and Cardiovascular Surgery, University Hospital RWTH Aachen, Pauwelsstrasse 30, 52074 Aachen, Germany; 2 Department of Infection Control and Infectious Diseases, University Hospital RWTH Aachen, Aachen, Germany

**Keywords:** Mediastinitis, Percutaneous Dilatational Tracheostomy (PDT), Cardiac surgery, Sternal Wound Infection (SWI), Cross-infection, Postoperative complication

## Abstract

**Background:**

The impact of percutaneous dilatational tracheostomy (PDT) on the development of post-median sternotomy wound infection (SWI) and mediastinitis is still controversial. We aimed to investigate the frequency of cross-infection and incidence of SWI after PDT.

**Methods:**

In a retrospective design, out of a total of 4100 procedures, all patients who had undergone median sternotomy and postoperative PDT were included from January 2010 to May 2013. For comparison of the pathogens isolated from SWIs, data from all patients who developed an SWI without a PDT during the aforementioned period were also analyzed. Demographical, pre-, peri- and post-operative data were compared. Microbiologic analysis from cultures of sternal and tracheal wounds was performed. Day and duration of tracheostomy were correlated to SWI occurrence.

**Results:**

Of the 265 patients who underwent a PDT, 25 (9.4 %) developed an SWI. In this cohort, identical pathogens were isolated from the tracheostomy and SWI in 36 % (9/25) of the patients. Of the pathogens isolated from the SWIs from the PDT + SWI group, 60 % were gram-positive bacteria, 20 % gram-negative bacteria and 20 % *Candida spp*. In the cross-infection group, the patients developed the following types of SWIs: 11.1 % CDC I, 55.6 % CDC II and 33.3 % mediastinitis (CDC III). The incidence of SWI in the group SWI + PDT was 9.4 % (9.4 % vs. 3.4 %, PDT + SWI and SWI_*w/oPDT*_, respectively, *p* = 0.0001). In group SWI_*w/oPDT*_, only 1.5 % (2/131 vs. 5/25; *p* = 0.001) *Candida spp* were isolated from SWI. The infection-related in-hospital mortality was high in groups PDT + SWI vs. SWI_*w/oPDT*_ (20 % vs. 0 %, respectively; *p* = 0.0001). The statistical analysis did not demonstrate any correlation between time of performing PDT and occurrence of SWI.

**Conclusions:**

There was a high incidence of microbial cross-infection from the PDTs to the sternal wounds in our study. We did not detect any correlation between the time of performing PDT and occurrence of SWI. According to our data, PDT seems to increase the incidence of SWI, especially caused by *Candida spp*., after cardiac surgery, which results in a prolonged hospital stay. Therefore, early antifungal prophylaxis after a PDT might be reasonable in high-risk patients on long-term mechanical ventilation if there is an impending SWI.

## Background

Sternal wound infection (SWI) and mediastinitis are devastating complications in cardiac surgery patients and are associated with high mortality rates between 10 and 50 % [1–4]. The incidence of deep sternal wound infection (DSWI) and mediastinitis have been reported to range between 0.16–3.20 % and 1–3 %, respectively [1, 3, 4]. The most common microbiological pathogens found in infected sternal wounds are gram-positive staphylococci (*Staphylococcus aureus* and coagulase-negative *staphylococci*) followed by gram-negative species [5–10]. *Candida spp*. were also isolated from SWIs and prevalent in patients on long-term mechanical ventilation.

In this retrospective study, we aimed to investigate the incidence of SWIs after percutaneous dilatational tracheostomies (PDTs), the frequency of cross-infection and if a PDT changed the microbial strains involved in an SWI.

## Methods

From January 2010 to May 2013, 4100 cardiac surgery procedures through median sternotomy were performed at our institution. All cardiac surgery patients who had undergone cardiac surgery with a full median sternotomy and required a postoperative PDT were included in this retrospective study.

**Fig. 1:**
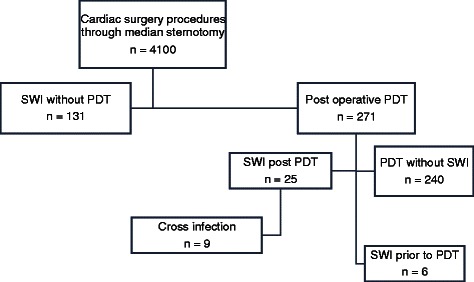
Classification of patients in groups

A PDT was performed within a monitored time window in 271 of 4100 patients undergoing full-sternotomy cardiac surgery in our unit. Of these 271 patients, six patients who developed SWI prior to PDT were excluded from the study. From the remaining 265 patients, 25 (9.4 %) developed an SWI or mediastinitis post-PDT (group PDT + SWI, *n* = 25). For comparison of the pathogens isolated from SWIs, data from all patients who developed an SWI without a PDT during the aforementioned period were also analyzed (group SWI_*w/oPDT*_, *n* = 131) (Fig. 1). EuroSCORE II and The Society of Thoracic Surgeons’ risk models (STS) scores were calculated for all patients. All patients with SWI were followed up routinely in our out patient clinic for 6 weeks after discharge from the hospital. The study protocol was cleared by the local ethical committee, Ethik-Kommission an der Medizinischen Fakultät der RWTH Aachen (KEK). Due to the retrospective character of this observational study, informed consent was waived by the local board at the Medical Faculty RWTH Aachen.

### Procedural routine

All the patients underwent a similar pre-operative assessment and variety of cardiac procedures using standard median sternotomy. All the patients undergoing cardiac surgery in our department routinely receive Mupirocin nasal ointment on the day before operation and a single shot of peri-operative prophylactic antibiotics with either ampicillin/sulbactam (Unacid®) or, if a patient has a penicillin allergy, clindamycin.

According to our institutional standard operating procedures, tracheostomies were performed in patients who failed extubation twice or when patients were not expected to be extubated within 10 days. All PDTs were performed by an experienced team of anesthesiologists in the ICU using the Frova technique (RÜSCH Tracheostomy, Teleflex Medical GmbH, Kernen, Germany).

### Data assessment

The following data were collected for all the patients: pre-operative, peri-operative and post-operative parameters, duration of intubation, duration of mechanical ventilation, microbiological findings from tracheal secretions, occurrence of SWI, all-cause in-hospital mortality and infection-related in-hospital-mortality, microbiological findings from sternal wound, common pathogens isolated from the sternal wound and tracheostoma, post-operative day on which PDT was performed, and the duration of ventilation through PDT.

Sternal wound infections and mediastinitis were classified according to the guidelines of the Centers for Disease Control and Prevention (CDC) [11] as follows: superficial sternal wound infection SSWI (CDC I), deep sternal wound infection DSWI (CDC II) and mediastinitis (CDC III). Aspirates from tracheal secretions were routinely collected and sent for microbiological analysis before or on the day a PDT was performed as well as the second or third day after the PDT procedure. In cases of infection, samples were taken at least two times a week.

An SWI was diagnosed by a clinical examination (signs of local infection, drainage of pus, fistulas and fever), computed tomography (CT) scans (retrosternal fluid collection and sternal dehiscence) and lab findings (leucocytosis and C-reactive protein). In all cases, the diagnosis was confirmed by microbiological findings. Wound swabs were taken before debridement and wound cleansing and sent for microbiological culture diagnostics. Once a mediastinal infection was evident, appropriate antibiotics were administered based on culture and sensitivity results.

### Statistics

Data analysis was performed with SPSS 20 (IBM, Chicago, IL, USA). Continuous variables were compared with means ± SD using Student´s *t*-test. Categorical variables were analyzed with a Chi-Square test or, if appropriate, Fisher’s exact test. Mortality at defined time points and incidence of SWIs were compared using a Chi Square test. Incidence of SWIs in relation to a categorized time point after a PDT procedure and duration of respiratory therapy were analyzed using Pearson’s chi-squared test (*χ*^2^). All *p*-values were reported as three digit numbers or with at least one non-zero digit. A *p*-value < 0.05 was considered statistically significant.

## Results

### Demographics

In group PDT + SWI, both the EuroScore II and STS scores indicated that most of the patients were high risk.

The groups PDT + SWI and SWI_*w/oPDT*_ differed significantly in many known risk factors (Table 1): mean body mass index (BMI), *p* = 0.034; peripheral arterial disease (PAD), *p* = 0.0005 and renal insufficiency, *p* = 0.029; pre-operative intra-aortic balloon pump (IABP), *p* = 0.024; and pre-operative inotrope therapy, *p* = 0.013. Group PDT + SWI had a higher mean EuroSCORE II score than group SWI_*w/oPDT*_ (18.81 vs. 4.0, respectively, *p* = 0.0001), a higher mean STS score for risk of mortality (6.5 vs. 2.2, respectively, *p* = 0.0001), and no significant difference in mean STS score for risk of DSWI (0.6 vs. 0.5, respectively, *p* = 0.231). Group SWI_*w/oPDT*_ included more patients who underwent CABG surgery than group PDT + SWI (71.8 % vs. 48.0 %, *p* = 0.033). There were no differences in perioperative variables between both groups. Only the mean cardiopulmonary bypass time (CPB) (*p* = 0.013) was significantly longer in group PDT + SWI.

**Table 1:** Demographics and clinical data

	PDT + SWI (*n* = 25)	SWI w/o PDT (*n* = 131)	*P*-values
Preoperative variables			
Mean age (±) SD	70 (±6.4)	66 (±11)	0.080
Mean BMI (±) SD	**26.8 (±3.93)**	**28.9 (±4.6)**	**0.034**
Female % (n)	36.0 (9)	44.3 (58)	0.511
Preop. LVEF < 34 % (n)	20.0 (5)	4.6 (6)	0.729
NYHA IV % (n)	36.0 (9)	14.5 (19)	0.420
Hypertension % (n)	88.0 (22)	93.9 (123)	0.385
DM % (n)	32.0 (8)	42.0 (55)	0.383
COPD % (n)	36.0 (9)	24.4 (32)	0.226
PAD % (n)	**48.0 (12)**	**14.5 (19)**	**0.0005**
Smoking % (n)	48.0 (12)	47.3 (62)	1.000
Recent MI % (n)	44.0 (11)	42.7 (56)	1.000
Renal insufficiency % (n)	**68.0 (17)**	**43.5 (57)**	**0.029**
Preop. IABP % (n)	**8.0 (2)**	**0**	**0.024**
Preop. Inotropes % (n)	**16.0 (4)**	**2.2 (3)**	**0.013**
Preop. Dialysis % (n)	4.0 (1)	0.7 (1)	0.295
Mean Euroscore II in % (±) SD	**18.8 (±15.6)**	**4.0 (±4.6)**	**0.0001**
Mean STS-score: Risk of Mortality % (±) SD	**6.5 (±4.3)**	**2.2 (±2.1)**	**0.0001**
Mean STS-score: Risk of DSWI % (±) SD	0.6 (±0.26)	0.5 (±0.4)	0.231
Operative procedures			
CABG % (n)	**48.0 (12)**	**71.8 (94)**	**0.033**
Comb. CABG + Valve % (n)	12.0 (3)	16.0 (21)	0.768
Valve surgery % (n)	8.0 (2)	1.5 (2)	0.120
Thoracic aorta proc. % (n)	16.0 (4)	4.6 (6)	0.055
VAD-Implantation % (n)	8.0 (2)	0.8 (1)	0.067
Re-do % (n)	8.0 (2)	5.3 (7)	0.637
Perioperative variables			
Mean Cross-clamp time (min)	58.6 (±44.0)	59.0 (±36.1)	0.961
Mean CPB time (min)	**133.8 (±59.5)**	**103.8 (±54.3)**	**0.013**
Postoperative Variables			
Re-thoracotomy (%)	20 (5)	9.2 (12)	0.153
Stroke (%)	**12 (3)**	**0**	**0.003**
Low cardiac output (%)	**20 (5)**	**0**	**0.0001**
Pneumonia % (n)	**68 (17)**	**13.7 (18)**	**0.0001**
Respir. Insufficiency % (n)	**84 (21)**	**19.0 (25)**	**0.0001**
Delirium % (n)	**56 (14)**	**22.9 (30)**	**0.001**
Post-op MI % (n)	0	1.5 (2)	1.000
Sepsis	**60 (15)**	**13.7 (18)**	**0.0001**
Mean vent. time in (h) (±) SD	**547 (±288)**	**21 (±37)**	**0.0001**
Mean number of packed RB cells (±) SD	**11 (±11)**	**3 (±2)**	**0.0001**
Mean days of ICU-stay (±) SD	**32 (±11)**	**7 (±8)**	**0.0001**
Mean LOS hospital (±) SD	40 (±14)	36 (±18)	0.294

Description of pre-, peri-, post-operative clinical characteristics, surgical techniques and intensive care unit stays

**Bold** writing indicates significant values

*BMI* body mass index (kg/m^2^), *LVEF* left ventricle ejection fraction, *NYHA* New York Heart Association, *COPD* chronic obstructive pulmonary disease, *DM* diabetes mellitus, *PAD* peripheral arterial disease, *Recent MI* recent myocardial infarction, *Preop* pre-operative, *IABP* intra-aortic balloon pump, *CABG* coronary artery bypass graft, *Other* other open heart surgery procedures, *CPB time* total cardiopulmonary bypass time (in min), *Valve* heart valve surgery, *VAD* ventricular assist device implantation, *Re-do* second heart surgery, *SD* standard deviation, *LOS* length of stay, *ICU* intensive care unit; *packed RB cells* packed red blood cell

### Infection rates and mortality

**Fig. 2:**
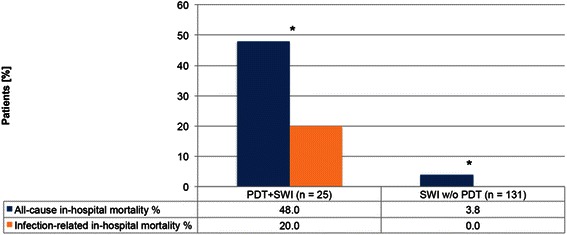
All-cause in-hospital mortality and infection-related in-hospital mortality. *p = 0.0001

During the study period, the overall incidence of SWI was 3.8 % (156/4100 cardiac surgery procedures with a full median sternotomy) with a low incidence of mediastinitis (0.56 %; 23/4100). In group PDT + SWI, the incidence of SWI was significantly higher than in group SWI_*w/oPDT*_ with 9.4 % versus 3.4 %, respectively; *p =* 0.0001 (Table 2). In both groups, most cases of SWI and mediastinitis were detected within the first 30 post-operative days (72 % in group PDT + SWI and 84 % in group SWI_*w/oPDT*_). In group PDT + SWI, five patients died because of mediastinitis-related septic multi-organ failure, compared to group SWI_*w/oPDT*_ in which no infection-related in-hospital mortality was detected (20 % vs. 0 %, respectively; *p* = 0.0001). All-cause in-hospital mortality was significantly higher in group PDT + SWI than in group SWI_*w/oPDT*_ (48 % vs. 3.8 %, respectively; *p* = 0.0001) (Fig. 2).

**Table 2:** Incidence of SWI in group PDT + SWI vs. group SWI w/o PDT

	PDT + SWI (*n* = 25)	SWI w/o PDT (*n* = 131)	*P*-Values
SWI % (n)	**9.4 % (25/265)**	**3.4 % (131/3829)**	**0.0001**
CDC I % (n)	**20 % (5)**	**1.5 % (2)**	
CDC II % (n)	**52 % (13)**	**86.3 % (113)**	
CDC III % (n)	**28 % (7)**	**12.2 % (16)**	

**Bold** writing indicates significant Values

*CDC* Centers for Disease Control and Prevention, *SWI* sternal wound infection

### Microbiological findings

**Fig. 3:**
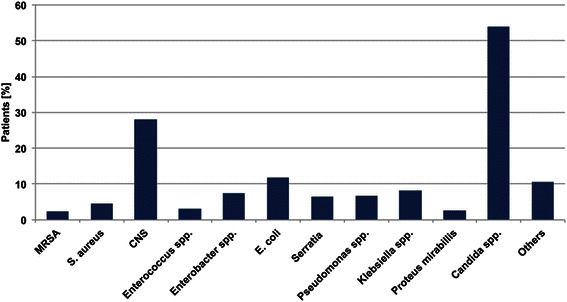
Bacteria isolated from tracheal secretions in group PDT + SWI. MRSA: Methicillin-resistant Staphylococcus aureus; CNS: Coagulase negative staphylococci; E. coli: Escherichia coli; others: described in Table 3

**Fig. 4:**
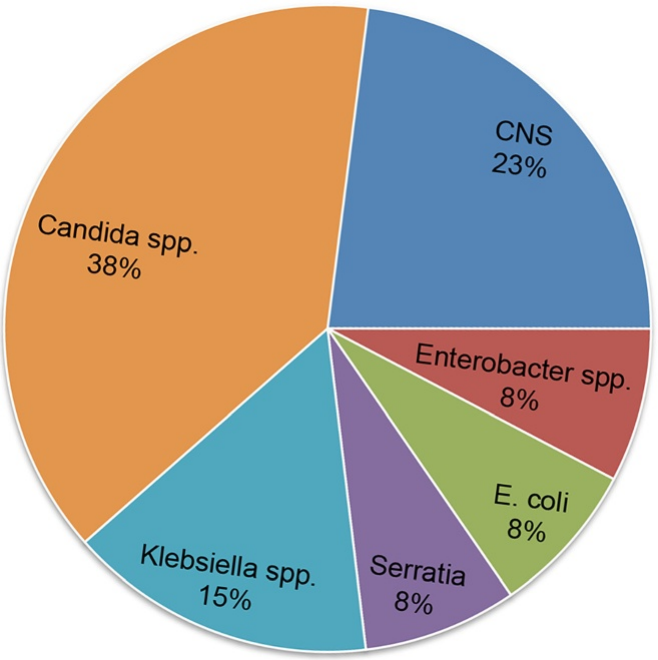
Common pathogens isolated from tracheostomas and sternal wounds. MRSA: Methicillin-resistant Staphylococcus aureus; CNS: Coagulase negative staphylococci; E. coli: Escherichia coli

Figure 3 demonstrates the microbiological pathogens isolated from tracheal secretions of the patients who received a PDT. In group PDT + SWI, identical pathogens were isolated from the tracheostomy and sternal wounds of nine (36 %) patients. The majority of patients with a post-operative SWI or mediastinitis had polymicrobial infections. The common pathogens isolated from the tracheal secretions and sternal wounds are shown in Fig. 4. In nine patients, the cross-infection SWIs were classified as follows: 11.1 % CDC I, 55.6 % CDC II and 33.3 % mediastinitis (CDC III). One patient with cross-infection died because of mediastinitis-related septic shock. The common cross-infection pathogen was *Candida albicans.*

Pathogens isolated from the SWI in groups PDT + SWI and SWI_*w/oPDT*_ are outlined in Table *3*. In our study, the incidence of *Candida spp*. infections was significantly higher (20 % vs. 1.5 %, *p* = 0.001) in group PDT + SWI than group SWI_*w/oPDT*_. However, the incidence of *S. aureus* infections (8 % vs. 29 %, *p* = 0.05) was significantly higher in group SWI_*w/oPDT*_ than group PDT + SWI.

**Table 3:** Pathogens isolated from SWIs

	PDT + SWI	SWI w/o PDT	*P*-values
*MRSA*	8 % (2)	4.6 % (6)	0.615
*Staphylococcus aureus*	**8 % (2)**	**29 % (38)**	**0.026**
*CNS*	32 % (8)	40 % (52)	0.516
*Enterococcus spp.*	12 % (3)	3.1 % (4)	0.082
*Enterobacter spp*	4 % (1)	2.3 % (3)	0.506
*Escherichia coli*	8 % (2)	8.5 % (11)	1.000
*Serratia marcescens*	4 % (1)	3.1 % (4)	0.587
*Pseudomonas spp.*	0	4.6 % (6)	0.590
*Klebsiella spp.*	12 % (3)	2.3 % (3)	0.052
*Proteus mirabilis*	0	4.6 % (6)	0.590
*Candida spp.*	**20 % (5)**	**1.5 % (2)**	**0.001**
Others	**0**	**14.6 % (19)**	**0.044**

**Bold** writing indicates significant values

*MRSA* methicillin-resistant staphylococcus aureus, *CNS* coagulase negative staphylococci; others: Citrobacter youngae, Stenotrophomonas maltophilia, Aspergillus fumigatus, Streptococcus agalactiae, Morganella morganii, Haemophilus influenzae, Citrobacter braakii, Citrobacter koseri, Saccharomyces cerevisiae, Acinetobacter gyllenbergii and Lactobacillus curvatus

### Incidence of SWI in relation to the time after PDT procedure and duration of respiratory therapy

**Fig. 5:**
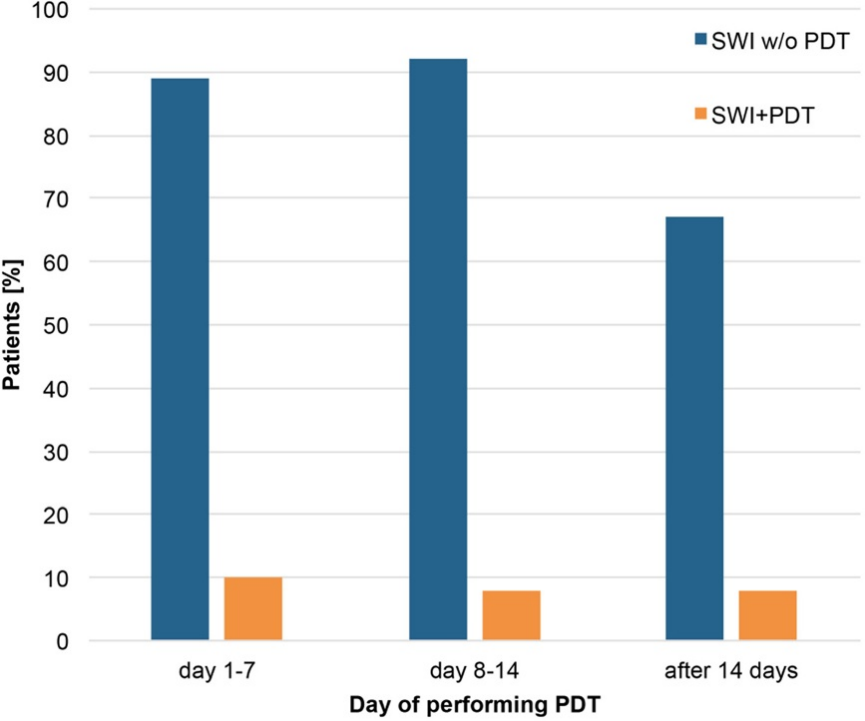
Correlation between time of performing PDT and SWI

**Fig. 6:**
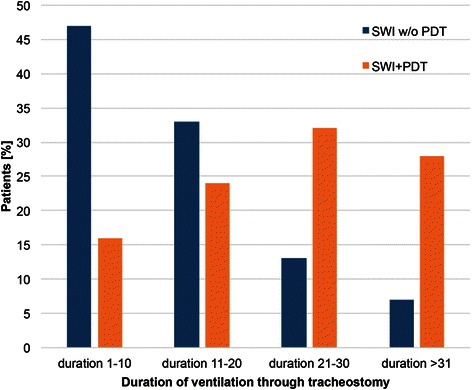
Correlation between duration of ventilation through PDT and SWI

During the first post-operative week, 207 patients received a PDT, and 20 patients in this group developed an SWI. During the second post-operative week, 49 patients underwent a PDT, and four developed an SWI. During the third week, a PDT was performed in nine patients; one developed an SWI (Fig. 5). After analyzing the relation of the incidence of SWIs to the categorized time point of PDTs and duration of respiratory therapy using Pearson’s Chi-squared test, no correlation was detected between the time of performing a PDT and SWI (*p* = 0.963, Fig. 6). However, there was a correlation (*p* = 0.0001) between the duration of ventilation through a PDT and occurrence of an SWI (Fig. 6).

## Discussion

### Clinical relevance of the bacterial strains

In group SWI_*w/oPDT*_, the most frequently isolated pathogens from SWIs were gram-positive *staphylococci* (CNS 40 %, followed by *S. aureus* 29 %). These findings are consistent with numerous studies [5–10]. In these studies, the incidence of fungi in SWI was reported to range between 1 and 5 %. In our group PDT + SWI, the incidence of *S. aureus* was significantly lower than group SWI_*w/oPDT*_. On the other hand, *Candida spp*. were isolated from 20 % of the SWIs after PDT. *Candida spp*. were also isolated from tracheal secretions in 53.6 % of the patients who had a PDT, as well as in 38 % of the patients who had cross-infection between the PDT and SWI. Five patients with an SWI due to *Candida spp*. contamination had the longest hospital stays (45 ± 15 days). One of them died during the first 30 POD from shock and multiorgan failure related to mediastinitis. The low incidence of S. aureus in SWI + PDT and subsequent high rate of candida spp. might be related to the fact that these patients already suffered a long term intensive care stay with multiple complications requiring long term antibiotics.

Our findings are consistent with the study from Modrau et al. [7]. They found that when cardiothoracic patients on mechanical ventilation are tracheally colonized with *Candida spp*., they have a high-risk for subsequently developing *Candida* DSWI. Additionally, patients who developed SWI with *Candida spp*. had a significantly higher mortality rates and longer ICU stays.

The patients who required a PDT were critically ill patients with postoperative complications such as pneumonia requiring long-term antibiotic therapy. Pittet et al. [12] identified the length of antibiotic therapy, severity of illness and degree of *Candida spp*. colonization as factors that predicted subsequent *Candida* infections. A Cochrane Review suggested that antifungal prophylaxis should be considered in non-neutropenic critically ill patients [13].

### Cross-infection

We found cross-infection in nine (36 %) patients with an SWI after a PDT. These findings may be controversial because several previous studies did not find any correlation between early PDT and SWIs [14–16]. Compared with an open surgical tracheostomy, a PDT causes less microbial contamination at the tracheostomy site [17, 18]. Whether the PDT technique prevents microbiological cross-infection of an adjacent sternal wound remains a controversial matter.

### Tracheostomy as a risk factor

Prolonged mechanical ventilation after cardiac surgery is an unfortunate adverse event occurring in patients suffering respiratory failure. This can be due to a variety of reasons, pneumonia being the most common.

Sun et al. [19] reported tracheostomy as an independent risk factor for SWI. On the other hand, cardiac surgery patients who require prolonged mechanical ventilation benefit from tracheostomy [20, 21] because of a reduced level of sedation and facilitated weaning from ventilator support, which results in a reduced incidence of ventilator-associated pneumonia [22, 23]. PDT has become a standard procedure in cardiac surgery patients who have a high risk for post-operative pulmonary complications and replaced open surgical tracheostomy [17, 24]. A PDT is easy to perform, safe cost-effective and can be performed at bedside [17, 24]. However, some authors state that a PDT does not increase the risk for an SWI following a median sternotomy, even when the PDT is carried out in the first post-operative week [16, 17, 25]. In accordance with Byhahn et al., Hubner et al. and Gaudino et al. [14, 15, 25], we found that there was no correlation between an early postoperative PDT and the development of SWI. From a clinical point of view, we strongly support Byhahn et al., Stamenkovic et al. and Gaudino et al. [14, 16, 25] who concluded that the indisputable advantages of early PDT far outweigh the potential risks of promoting an SWI. However, according to our findings, early antifungal prophylaxis when an SWI is imminent might be reasonable in high-risk patients with a PDT to avoid dire complications of a candida wound infection.

### Limitations of study

One of the main limitations of the presented study is its retrospective design, although prospective designs can also be descriptive if there are ethical and practical limitations. Additionally, SWI registries would enhance data collection and aggregation and could be used to gain new insights on the topic.

Due to the small number of patients with simultaneous SWI and PDT, it was not possible to identify risk factors for SWIs caused by cross-infection.

As a matter of fact, PDT is limited to critically ill patients. Therefore a comparison of patients suffering SWI with or without PDT leads to a heterogeneous distribution of preoperative risk factors, limiting comparability of outcomes and postoperative characteristics. We emphasized this by highlighting the preoperative differences between groups in Table 1.

In the routine microbiological tests of tracheal secretions in our laboratory, not all bacteria of normal upper airways flora were directly identified. Therefore, they were not mentioned separately in the microbiological reports. Many pathogens belonging to normal upper airway flora are potential pathogens that can cause an SWI. Therefore, the rate of cross-infection could be higher than what we could detect.

## Conclusion

The incidence of microbial cross-infection from a PDT to a sternal wound in our study was high. We could not detect any correlation between the time of performing a PDT and occurrence of an SWI. According to our data, PDT seems to increase the incidence of SWI, especially caused by *Candida spp*., after cardiac surgery, which results in a prolonged hospital stay. In high-risk patients on long-term mechanical ventilation early antifungal prophylaxis after a PDT might be reasonable as soon as an SWI is suspected in order to mitigate dire complications of a candida SWI.

## Abbreviations

BMI: mean body mass index
CABG: coronary artery bypass grafting
CDC: Centers for Disease Control and Prevention
CNS: coagulase-negative staphylococci
CPB: cardiopulmonary bypass time
CT: computed tomography
DSWI: deep sternal wound infection
E. coli: escherichia coli
IABP: intra-aortic balloon
PAD: peripheral artery disease
PDT: percutaneous dilatational tracheostomy
spp.: species
S. aureus: staphylococcus aureus
SSWI: superficial sternal wound infection
STS: The Society of Thoracic Surgeons’ risk models
SD: standard deviation
SWI: sternal wound infection
