# Erratum to: Does percutaneous dilatational tracheostomy increase the incidence of sternal wound infection - a single center retrospective of 4100 cases

**DOI:** 10.1186/s13019-016-0423-1

**Published:** 2016-02-08

**Authors:** Lachmandath Tewarie, Rachad Zayat, Helga Haefner, Jan Spillner, Andreas Goetzenich, Rüdiger Autschbach, Ajay Moza

**Affiliations:** Department of Thoracic and Cardiovascular Surgery, University Hospital RWTH Aachen, Pauwelsstrasse 30, Aachen, 52074 Germany; Department of Infection Control and Infectious Diseases, University Hospital RWTH Aachen, Aachen, Germany

Unfortunately, after publication of this article [1] it was noticed that the Authors’ Contributions section contained a mistake. The correct text can be seen below:

## Authors’ contributions

LT and RZ designed the study and developed the database. LT and RZ performed the statistical analyses and drafted the manuscript. RZ wrote the manuscript. AG critically revised the statistical analysis, the study design and the manuscript. AM critically revised the manuscript in cooperation with the co-authors and interpreted the data. HH critically revised the microbiological findings. RA, as the department chair, supported this study and participated in designing the study. RA and JS critically revised the manuscript. RZ collected the patient data. All the authors have read and approved the submitted version of the manuscript.
